# Insights into ALG3-CDG: A case study combining glycan profiling and genetic analysis

**DOI:** 10.1016/j.ymgmr.2025.101263

**Published:** 2025-09-30

**Authors:** Rebeka Kodríková, Zuzana Pakanová, Maroš Krchňák, Veronika Krajčovičová, Anna Šalingová, Katarína Skalická, Miriam Kolníková, Peter Baráth, Marek Nemčovič

**Affiliations:** a Center of Glycomics, Institute of Chemistry, Slovak Academy of Sciences, Dúbravská cesta 9, 841 04 Bratislava, Slovakia; b Center for Inherited Metabolic Disorders, National Institute of Children's Diseases, Limbová 1, 833 40 Bratislava, Slovakia; c Laboratory of Clinical and Molecular Genetics, National Institute of Children's Diseases, Limbová 1, 833 40 Bratislava, Slovakia; d Department of Pediatric Neurology, National Institute of Children's Diseases, Limbová 1, 833 40 Bratislava, Slovakia

**Keywords:** ALG3-CDG, Congenital disorders of glycosylation, RapiFluor, LC-MS, Mass spectrometry

## Abstract

Congenital disorders of glycosylation (CDG) are a group of rare metabolic disorders caused by the defects in the glycosylation pathways of biomacromolecules leading to altered glycoprofiles in affected individuals. In this case study, we present a 3-year-old Slovak male patient with developmental delay, hearing impairment, epilepsy, microcephaly, facial dysmorphism, corpus callosum dysgenesis, and cardiac abnormalities. To elucidate the underlying cause, we performed LC-ESI-MS analysis of RapiFluor-labelled *N-*glycans released from blood serum glycoproteins. The results revealed an abnormal *N*-glycan profile, characterized by an increased relative abundance of truncated mannosylated structures (Hex3HexNAc2 and Hex4HexNAc2) and a decreased presence of higher-order mannose structures (Hex6-8HexNAc2). A molecular analysis was also conducted. Whole exome sequencing confirmed a diagnosis of ALG3-CDG with compound heterozygous variants: c.165C > T (p.Gly55=) and c.1060C > T (p.Arg354Cys) in the *ALG3* gene, encoding alpha-1,3-mannosyltransferase in the endoplasmic reticulum. This presented case highlights the importance of glycan profiling and genetic analysis in diagnosing congenital disorders of glycosylation, facilitating early intervention and management.

## 1 Introduction

Congenital disorders of glycosylation (CDG) are a group of rare inherited metabolic disorders developing due to the incorrect glycosylation pathway of the proteins and lipids leading to their hypoglycosylation. In recent years, the number of CDG cases has increased due to advancements in diagnostics and improved collaboration between clinicians and researchers. To date, approximately 160 subtypes of CDGs have been identified, with the majority resulting from defects in *N-*glycosylation of proteins occurring in the endoplasmic reticulum (synthesis of lipid-linked oligosaccharide precursor - Glc3Man9GlcNAc2-PP-Dol) and Golgi apparatus (trimming and processing of glycans already bound to a protein) [1]. In general, CDGs are multi-systemic disorders characterized by a diverse range of clinical symptoms, frequently affecting the central nervous system, muscular system, cognitive functions and individual development, while hepatic and gastrointestinal difficulties are also commonly observed [2,3].

ALG3-CDG (OMIM 601110) is an autosomal recessive disorder caused by a mutation in the *ALG3* gene (chromosome 3q27) encoding alpha-1,3-mannosyltransferase in the endoplasmic reticulum (ER), an enzyme responsible for adding 6th mannose residue in an alpha-1,3 linkage to Man5GlcNAc2-PP-Dol. So far, around 43 patients with ALG3-CDG have been reported, while most of the cases are caused by missense/nonsense variants (HGMD® Professional, accessed on 14th of November 2024) with a few nonsense and splice junction variants [4,5]. In patients with ALG3-CDG, increased levels of asialo- and disialo-transferrin isoforms, characteristic of CDG type I, were observed through isoelectric focusing of serum transferrin (IEF-Tf) [[6], [7], [8]]. As with other CDGs, ALG3-CDG is also a multisystem disease. Patients suffer from combinations of various clinical symptoms, such as developmental delay, neurological disabilities (epilepsy, seizures, microcephaly, cerebral and cerebellar atrophy, agenesis of corpus callosum), facial dysmorphism, cardiac defects, visual impairment and muscular abnormalities [5,9,10]. Currently, the treatment for ALG3-CDG is only symptomatic, supporting clinical manifestations of the patients.

In this work, we present a male patient with biallelic variants in the *ALG3* gene revealed by whole exome sequencing (WES) and selective screening test (IEF-Tf). On the basis of the positive results of IEF-Tf, structural analysis of fluorescently labelled *N*-glycans in the patient's serum was performed using HILIC-FLD-ESI-MS analysis.

## 2 Methods

### 2.1 Isoelectric focusing of transferrin

Isoelectric focusing of serum transferrin (IEF-Tf) was performed as described previously in Kodríková et al. [11]. Briefly, the analysis used a PhastSystem (GE Healthcare) to separate serum proteins by pI on a PhastGel (pH 5–8). The transferrin (Tf) isoforms were then immuno-fixed with anti-Tf antibodies (Dako), stained with Coomassie Blue, and densitometrically quantified using GelAnalyzer 2010a for the percentual evaluation of individual Tf isoforms.

### 2.2 Whole exome sequencing and sanger sequencing

Genomic DNA was isolated from the blood of the patient and parents using a NucleoSpin Blood QuickPure (Macherey-Nagel, Duren, Germany). WES was conducted on the Nextseq 550 platform (Illumina, San Diego, USA) using the Illumina DNA Prep with Exome 2.5 Enrichment protocol and the Illumina Exome Panel (Twist Bioscience, San Francisco, USA) for library preparation. Raw sequencing data were processed using the NextGENe v2.4.2 tool and Geneticist Assistant software (version 1.8) (Softgenetics, State College, USA). Genomic regions of genes associated with hereditary metabolic disorders were evaluated and identified variants automatically annotated by the Geneticist Assistant software (version 1.8) based on data integrated from the following bioinformatic and in-silico prediction tools and population databases: SIFT, PolyPhen2, LRT, Mutation Taster, PhyloP, GERP++, SiPhy, ExAC, GnomAD, Exome Variant Server. Variants were further comprehensively evaluated and compared using literature, genotype-phenotype correlations, and public databases (ClinVar, Varsome, Franklin).

Identified variants were confirmed by PCR and Sanger sequencing. Segregation analysis was performed in the family (parents). For the c.165C > T (p. Gly55=) variant, the forward primer used was 5′-GAGCCAGAGGGCTATGTGA-3′ and the reverse primer was 5′-CCCCAACAGGAACTCCCTA-3′. For the c. 1060C > T (p.Arg354Cys) variant, the forward primer used was 5′-GCGGAACCTAAGTGTCGAAG-3′ and the reverse primer was 5′-GCAATTGACGAGTGAGTGGA-3′. DNA amplification was performed with MyTaq DNA Polymerase (Bioline, Cincinnati, USA) and amplified PCR products were sequenced using the BigDyeTerminator v3.1 Cycle Sequencing Kit (Applied Biosystems, Waltham, USA) with the PCR primers as sequencing primers on the ABI 3130xl Genetic Analyzer (Applied Biosystems, Waltham, USA). Data generated by Sanger sequencing were analyzed using ChromasPro (v1.5).

### 2.3 N-glycan analysis by LC-MS

*N-*glycans from serum were released and fluorescently labelled using GlycoWorks RapiFluor-MS *N-*glycan kit (Waters, Milford, USA) according to the manual. The serum samples were prepared in three technical replicates. Briefly, 1 μL of serum was diluted, heat-denatured, deglycosylated with PNGase F, and labelled with RapiFluor-MS. Subsequently, the glycans were cleaned on a HILIC μElution plate, vacuum-dried, reconstituted, and injected into an LC-FLD-MS system. The glycans were separated on the Dionex UltiMate 3000 UPLC system with ACQUITY UPLC BEH Amide Column. For detection, a Dionex fluorescence detector and Orbitrap Elite MS with positive ion mode were used. The data was analyzed using Freestyle software (v. 1.8.63.0, Thermo Scientific, Waltham, USA). Relative abundances of individual glycans were calculated from their respective extracted ion chromatogram (EIC) peak areas normalized to the dominant peak area (Hex5HexNAc4NeuAc2). The data were tested using a two-tailed, two-sample unequal variance *t*-test (α = 0.05). The datasets and detailed experimental method are deposited in the MS data repository for glycomics (GlycoPOST) [12] under project identification no. GPST000542.

## 3 Results

### 3.1 Clinical manifestation and molecular analysis

**Fig. 1:**
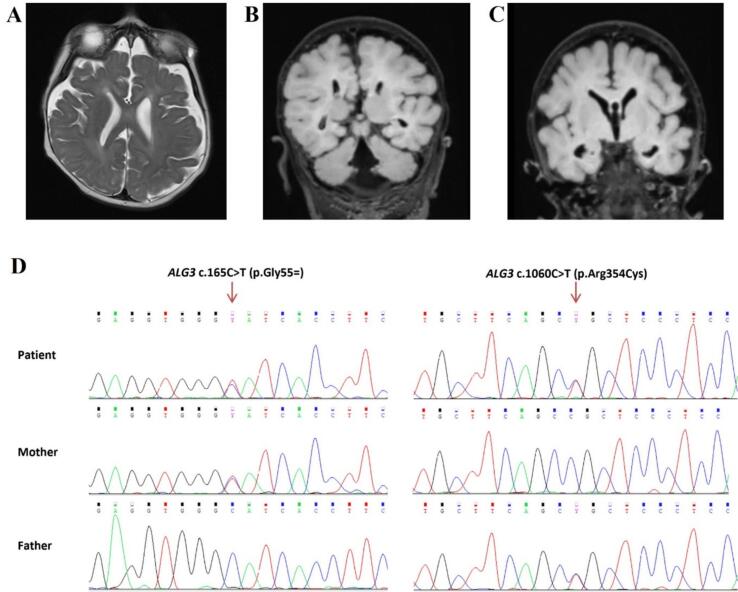
Brain MRI findings on A) T2-weighted (T2W) and FLAIR images (B, C) revealed delayed myelination of white matter, diffuse cortico-subcortical atrophy of supratentorial brain tissue, dysgenesis of the corpus callosum, and a dilated cisterna magna in a 9-months of age. D) Sequence chromatograms from Sanger sequencing showing variants identified in the patient and his parents. The patient carries compound heterozygous variants, c.165C > T (p. Gly55=) and c.1060C > T (p.Arg354Cys), in the *ALG3* gene. The mother is heterozygous for the c.165C > T (p. Gly55=) variant, while the father is heterozygous for the c.1060C > T (p.Arg354Cys) variant.

The patient is a 3-year-old boy from Slovakia, born prematurely at 33 weeks due to a high-risk pregnancy, weighing 2550 g at birth. Parents were asymptomatic and consanguinity was denied. His prenatal brain MRI revealed incomplete development of the corpus callosum and the absence of the cavum septi pellucidi. After the birth, the patient was placed on artificial pulmonary ventilation and received surfactant therapy. Psychomotor development was delayed. In the newborn age, the first epileptic seizures were observed and treated with ASM (levetiracetam). At 6 months of age, a series of spasms appeared, together with multiple epileptic seizures leading to hospitalization at the neurological department. The patient was diagnosed with a bilaterally perceived hearing disorder and hypertonus, microcephaly, and facial dysmorphia. Follow-up MRI scans indicated delayed demyelination of the white matter, acute changes in capsula interna with interference in the mesencephalon and pons medially dorsally bilaterally, also present diffuse cortico-subcortical atrophy of brain tissue supratentorially, cavum septi pellucidi, corpus callosum dysgenesis, enlarged cisterna magna (Fig. 1A, B, and C). Cardiological examination revealed atrial and ventricular defects. Based on biochemical laboratory tests, B_12_ hypovitaminosis was detected.

WES results revealed the presence of compound heterozygous variants, c.165C > T (p. Gly55=) and c.1060C > T (p.Arg354Cys), in the *ALG3* gene (NM_005787.5). Sanger sequencing confirmed the presence of these variants and revealed that the first variant was inherited from the mother, and the second from the father (Fig. 1D).

According to ACMG guidelines, the synonymous variant c.165C > T (p.Gly55=) in the *ALG3* gene was classified as pathogenic [13]. This substitution creates a cryptic donor splice site and has been described in several publications associated with congenital glycosylation disorder characterized by clinical manifestations such as epilepsy, dysmorphic changes, growth retardation, and hemangioma [8,[14], [15], [16], [17]]. ClinVar contains an entry for this variant (Variation ID: 2128). This variant (rs387906273) is present in the population database at a frequency of 0,00096 % (European/non-Finnish, gnomAD). *In silico* prediction tool suitable for splice region alterations predict a pathogenic effect of this substitution on protein structure and function (SpliceAI). The CADD score for this variant is 12.3, which falls within the range of variants considered significant and potentially pathogenic.

The missense variant c.1060C > T (p. Arg354Cys) in the *ALG3* gene was classified as likely pathogenic^13^. The substitution of the basic and polar arginine with the neutral and slightly polar cysteine is located in a phylogenetically conserved amino acid residue within a functional domain of ALG3 (Q92685, UniProt). ClinVar contains an entry for this variant (Variation ID: 2734602). This variant (rs762510540) is present in the population database at a frequency of 0,0035 % (European/non-Finnish, gnomAD). The CADD score for this variant is 32.0, which is consistent with a highly deleterious variant (UniProt).

### 3.2 Glycomic analysis of serum transferrin and protein N-glycoprofile

**Fig. 2:**
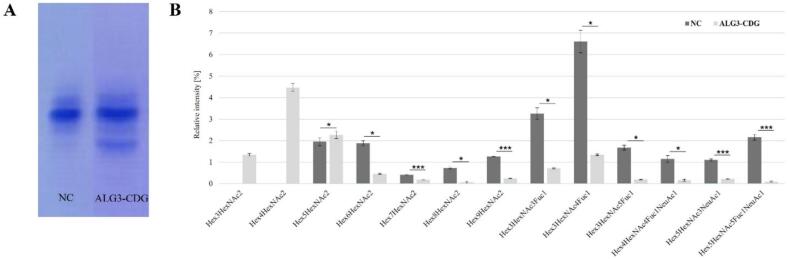
Glycomic analysis. A) Isoelectric focusing of serum transferrin of negative control (NC) and patient's serum transferrin with a result suggesting CDG-type I B) Relative quantification of individual *N-*glycans in serum of ALG3-CDG patient and negative control (NC). *p < 0.05, *** *p* < 0.001.

The isoelectric focusing of serum transferrin indicated a type I congenital disorder of *N-*glycosylation. The observed increase in asialo- and disialo- transferrin levels, along with a decrease in the tetrasialylated Tf isoform, suggested a glycosylation defect occurring in the endoplasmic reticulum (Fig. 2A). As isoelectric focusing of transferrin does not provide detailed information about specific subtypes or alterations in the overall N-glycoprofile, since the N-glycans bound to transferrin are primarily of the complex type, we performed mass spectrometry analysis.

LC-MS analysisof the patient's total blood serum glycoproteins, performed using HILIC-FLD-ESI-MS, revealed an abnormal *N-*glycoprofile compared to negative control. We selected 35 representative *N-*glycan structures to calculate their individual relative intensities. The major changes were observed in glycobiomarkers for ALG3-CDG - Hex3HexNAc2 and Hex4HexNAc2 glycans, which were absent (non-quantified) in the negative control sample. In contrast, the levels of higher oligomannose *N-*glycans (Hex6HexNAc2 through Hex9HexNAc2) were reduced, with Hex8HexNAc2 showing a significant 14.8-fold. The overall fucosylation of the identified *N-*glycans was decreased in the patient's sample, together with the reduced sialylation of glycoproteins. The relative abundances of the selected *N-*glycan structures showing notable changes are presented in Fig. 2B, while a comprehensive summary of all identified *N-*glycans and their relative abundances is provided in Table 1.

**Table 1:** Summary of the relative abundance of *N-*glycan structures identified in negative control and ALG3-CDG patient analyzed using LC-MS.

	Summary structure	Negative control (*n* = 3)			ALG3 patient (n = 3)			*p*-value
Fucosylated	Hex3HexNAc2Fuc1	0.86	±	0.123	0.11	±	0.006	<0.05
	Hex3HexNAc3Fuc1	3.26	±	0.271	0.72	±	0.026	<0.05
	Hex3HexNAc4Fuc1	6.61	±	0.523	1.34	±	0.035	<0.05
	Hex3HexNAc5Fuc1	1.67	±	0.113	0.19	±	0.015	<0.05
	Hex4HexNAc3Fuc1	1.41	±	0.007	0.35	±	0.199	<0.05
	Hex4HexNAc4Fuc1	9.76	±	0.542	2.47	±	0.043	<0.05
	Hex4HexNAc5Fuc1	2.30	±	0.126	0.29	±	0.021	<0.001
	Hex5HexNAc4Fuc1	6.04	±	0.123	3.01	±	0.066	<0.001
	Hex5HexNAc5Fuc1	0.72	±	0.060	0.19	±	0.012	<0.05
Oligomannose	Hex3HexNAc2	NQ			1.34	±	0.055	–
	Hex4HexNAc2	NQ			4.47	±	0.183	–
	Hex5HexNAc2	1.95	±	0.185	2.27	±	0.175	>0.05
	Hex6HexNAc2	1.88	±	0.109	0.46	±	0.021	<0.05
	Hex7HexNAc2	0.41	±	0.016	0.18	±	0.011	<0.001
	Hex8HexNAc2	0.73	±	0.041	0.05	±	0.040	<0.05
	Hex9HexNAc2	1.26	±	0.006	0.25	±	0.010	<0.001
Hybrid	Hex3HexNAc4	0.29	±	0.029	0.02	±	0.009	<0.05
	Hex3HexNAc5	0.35	±	0.017	0.06	±	0.008	<0.001
	Hex4HexNAc3	0.20	±	0.020	0.28	±	0.038	<0.05
	Hex4HexNAc4	0.63	±	0.075	0.16	±	0.034	<0.05
	Hex4HexNAc5	0.42	±	0.029	0.04	±	0.008	<0.001
	Hex5HexNAc4	0.82	±	0.019	0.67	±	0.022	<0.05
Sialylated	Hex4HexNAc3NeuAc1	2.47	±	0.096	3.39	±	0.093	<0.001
	Hex4HexNAc3Fuc1NeuAc1	0.37	±	0.036	0.39	±	0.037	>0.05
	Hex4HexNAc4NeuAc1	0.97	±	0.010	0.82	±	0.008	<0.001
	Hex4HexNAc4Fuc1NeuAc1	1.15	±	0.156	0.15	±	0.047	<0.05
	Hex5HexNAc3NeuAc1	1.10	±	0.049	0.22	±	0.021	<0.001
	Hex5HexNAc4NeuAc2	25.24	±	0.722	22.6	±	0.468	<0.05
	Hex5HexNAc4Fuc1NeuAc1	11.47	±	0.353	8.08	±	0.394	<0.001
	Hex5HexNAc4Fuc1NeuAc2	12.85	±	0.960	15.05	±	0.572	<0.05
	Hex5HexNAc5Fuc1NeuAc1	2.16	±	0.131	0.09	±	0.023	<0.001
	Hex6HexNAc3NeuAc1	0.75	±	0.031	0.16	±	0.017	<0.001
	Hex6HexNAc5NeuAc3	10.73	±	1.085	6.65	±	0.177	<0.05
	Hex6HexNAc5Fuc1NeuAc3	4.07	±	0.728	5.44	±	0.183	>0.05
	Hex7HexNAc6NeuAc4	0.44	±	0.069	0.50	±	0.078	>0.05

The values were calculated from EIC peak areas and peak intensities are relative to the highest peak (%) ± 1 SD. Both samples, negative control and ALG3-CDG patient, were analyzed in three technical replicates.

## 4 Discussion

In this study, we report a case of a 3-year-old Slovak patient who was identified with two compound heterozygous variants through whole exome sequencing, leading to ALG3-CDG, a rare metabolic multisystem disorder caused by deficient activity of alpha-1,3-mannosyltransferase. The patient's diagnosis was predicted based on clinical symptoms, a positive result from the selective screening test (IEF-Tf) for congenital disorders of glycosylation, and a comprehensive characterization of the *N-*glycoprofile (HILIC-FLD-ESI-MS) of the patient's blood serum.

The proteins of individuals with CDG type I are usually under-glycosylated or glycosylated incompletely, affecting their function and causing pathological multisystemic difficulties. The presented patient suffers from incomplete development of corpus callosum, microcephaly, seizures, and facial dysmorphism which are typical clinical symptoms characteristic of the patients with ALG3-CDG [10]. In addition to these features, the patient also presents with hearing impairment, hypertonia and brain tissue atrophy as was described in a Czech female patient with compound heterozygous variants in *ALG3* gene [5]. On the other hand, vitamin B12 deficiency has not been reported in previously described ALG3-CDG cases in the literature, thus is may be consider as a novel clinical feature. The standard screening test, isoelectric focusing of serum transferrin, detected type I CDG through a positive result characterized by elevated levels of asialo- and disialo- transferrin, similar to findings in other individuals with ALG3-CDG [9].

Further *N-*glycoprofile analysis of overall protein glycosylation and molecular distinguishing of the CDG subtype was performed. WES revealed two compound heterozygous variants, c.165C > T (p. Gly55=) and c. 1060C > T (p. Arg354Cys) in the *ALG3* gene, encoding protein alpha-1,3-mannosyltransferase. This combination of the variants has been already described [17] and both of the variants have been reported in patients with ALG3-CDG in compound heterozygosity with other variants [5,17,18]. In ALG3-CDG, deficient alpha-1,3-mannosyltransferase activity disrupts lipid-linked oligosaccharide (LLO) synthesis in the ER, leading to incomplete glycan structures like Dol-PP-GlcNAc2-Man5. These truncated LLOs result in the transfer of *N-*glycans to proteins, which may escape ER-associated quality control systems (unclear mechanism), causing incorrect folding and impaired glycoprotein function. These aberrant *N-*glycan structures, detectable in the patient's serum, serve as biomarkers for ALG3-CDG and can be identified through glycomic analysis [19]. Consistent with the findings by Chen et al. [19] described in their work, we observed increased intensities of the small oligomannose glycans, mainly Hex3HexNAc2 and Hex4HexNAc2. Such abnormal *N-*glycans were not detected in negative control by the presented LC-MS approach. Additionally, we would expect an accumulation of the Hex5HexNAc2 intermediate as its accumulation was also reported in previously described ALG3-CDG patients; however, its level was only slightly increased in the presented patient. Similar to other reported cases of ALG3-CDG, the level of Hex9HexNAc2 glycan was significantly reduced in the *N-*glycoprofile of the individual [[20], [21]]. Furthermore, the reduction in overall fucosylation could reflect decreased IgG levels, a phenomenon previously reported in some CDG subtypes [22]. As CDGs in general, are rare disorders, the small sample sizes in studies limit the statistical power, thus our conclusions are supported also by their consistency with previously published profiles. As type I CDGs are characterized by the accumulation of oligomannose precursors, we compared our patient's N-glycoprofile with that of other type I CDGs. A comparable *N-*glycoprofile pattern with changes in small oligomannose *N-*glycans, including increased levels of Hex3HexNAc2 and Hex4HexNAc2, may be detected in patients with other type I congenital disorders of glycosylation such as PMM2-CDG and MPI-CDG. However, ALG3-CDG can be distinguished from the PMM2-CDG and MPI-CDG by the presence of the tetrasaccharide (GlcNAc2Gal1Sial1), serving as the glycobiomarker for this CDG subtypes and is absent in individuals with ALG3-CDG [19,23]. Another CDG type I with an aberrant oligomannose N-glycosylation is ALG1-CDG. However, a pentasaccharide with Neu5Ac₁Gal₁Fuc₁GlcNAc_2_structure has been identified as a glycobiomarker, distinguishing it from other type I CDGs, such as presented ALG3-CDG [24]. Another subtype showing changes in the N-glycoprofile is ALG11-CDG. In previously reported patients, increased levels of the lipid-linked oligosaccharide precursors Hex3HexNAc2-PP-dolichol and Hex4HexNAc2-PP-dolichol were detected in fibroblast cell lines, compounds that are normally undetectable [25,26]. In some reported cases of ALG3-CDG, elevated levels of the glycan Hex5HexNAc2 were observed. This alternation is also increased in ALG12-CDG, together with Hex6HexNAc2, which distinguishes this subtype from ALG3-CDG [27]. Abnormal glycoprofile with the accumulation of the small oligomannose *N-*glycans was observed also in the plasma and fibroblasts of the female patient with ALG9-CDG in Davis et al. work [28]. The patient's *N-*glycoprofile showed a significant increase in Hex4HexNAc2-Hex6HexNAc2 and a reduction of the higher oligomannose *N-*glycans - Hex7HexNAc2-Hex9HexNAc2. *N-*glycan with the Hex3HexNAc2 structure was not detected, which differentiates the ALG9-CDG glycoprofile from that of ALG3-CDG [28]. Furthermore, in ALG2-CDG, alterations in small oligomannose N-glycans have not yet been reported; however, in theory, accumulation of truncated N-glycans could occur.

The rarity and diverse clinical manifestations of congenital disorders of glycosylation make their diagnosis challenging. An accurate detection is usually based on a multidisciplinary approach, involving collaboration among clinicians, diagnosticians, and chemical analysts, while glycobiomarkers can be instrumental in identifying distinct CDG subtypes. Furthermore, mass spectrometry–based N-glycoprofiling holds promise not only for enhancing diagnostic accuracy but also for supporting disease monitoring and therapy evaluation through the assessment of N-glycan changes.

## 5 Conclusions

The presented patient with compound heterozygous variants in the *ALG3* gene, causing a rare disorder, ALG3-CDG, showed abnormal changes in *N-*glycan abundance in the blood serum, similar to that of previously diagnosed patients with different variants. The *N-*glycoprofile is characterized by the accumulation of the small oligomannose *N-*glycans, serving as potential glycobiomarkers.

## Funding

This work was funded by the EU NextGenerationEU through the Recovery and Resilience Plan for Slovakia under the project No. 09I03-03-V04-00681; and 10.13039/501100006109VEGA 2/0054/25.

## Patient contest statement

An individual involved in the study provided written informed consent.

## Ethics statement

This case report was conducted in accordance with the ethical principles outlined in the Declaration of Helsinki and was approved by the Ethics Committee of the National Institute of Children's Diseases.

## CRediT authorship contribution statement

**Rebeka Kodríková:** Writing – original draft, Visualization, Validation, Data curation, Conceptualization. **Zuzana Pakanová:** Writing – review & editing, Writing – original draft, Funding acquisition. **Maroš Krchňák:** Writing – review & editing, Visualization, Validation, Methodology, Data curation. **Veronika Krajčovičová:** Resources, Methodology, Data curation. **Anna Šalingová:** Resources, Methodology, Data curation. **Katarína Skalická:** Resources. **Miriam Kolníková:** Investigation. **Peter Baráth:** Writing – review & editing. **Marek Nemčovič:** Writing – review & editing, Visualization, Funding acquisition, Conceptualization.

## Declaration of competing interest

The authors declare no conflicts of interest.

## Data Availability

Data will be made available on request.
